# A Novel ECG Eigenvalue Detection Algorithm Based on Wavelet Transform

**DOI:** 10.1155/2017/5168346

**Published:** 2017-05-17

**Authors:** Ziran Peng, Guojun Wang

**Affiliations:** ^1^School of Information Science and Engineering, Central South University, Changsha, Hunan Province 410083, China; ^2^Hunan Vocational College of Commerce, Changsha, Hunan Province 410205, China; ^3^School of Computer Science and Educational Software, Guangzhou University, Guangzhou, Guangdong Province 510006, China

## Abstract

This study investigated an electrocardiogram (ECG) eigenvalue automatic analysis and detection method; ECG eigenvalues were used to reverse the myocardial action potential in order to achieve automatic detection and diagnosis of heart disease. Firstly, the frequency component of the feature signal was extracted based on the wavelet transform, which could be used to locate the signal feature after the energy integral processing. Secondly, this study established a simultaneous equations model of action potentials of the myocardial membrane, using ECG eigenvalues for regression fitting, in order to accurately obtain the eigenvalue vector of myocardial membrane potential. The experimental results show that the accuracy of ECG eigenvalue recognition is more than 99.27%, and the accuracy rate of detection of heart disease such as myocardial ischemia and heart failure is more than 86.7%.

## 1. Introduction

ECG can record the physiological states of the heart and cardiovascular system in a real-time manner, and thus it is widely used for the detection and diagnosis of clinical heart disease [1]. ECG eigenvalue automatic detection can rapidly and accurately detect heart diseases [2]. Currently, ECG eigenvalue detection is based on multiple algorithms: the envelope analysis technique can effectively decompose complex signals into single component signals, which are typically empirical mode decomposition (EMD) and local mean decomposition (LMD). EMD is an adaptive signal decomposition method, the data from high frequency to low frequency decomposition into a series of intrinsic mode function (IMF) and a margin. Lahmiri and Boukadoum proposed A Weighted Bio-Signal Denoising Approach Using EMD in [3], which shows some advantages in ECG denoising. LMD solves the problem of endpoint effect of EMD method to a certain extent. However, both LMD and EMD belong to the recursive model, which have the problems of modal aliasing [4], end effect, being sensitive to noise and sampling, and difficulty in separating similar frequency components. But there is a problem caused by EMD [5]: in the background of bad noise, IMF will be submerged in the background of noise that leads to missing the signal characteristic component. Variational mode decomposition (VMD) solved this problem by transforming modal estimates into variational problems [6, 7].

The above methods are suitable for analyzing and dealing with aperiodic mutational signals [8]. If the periodic signals such as ECG are used to calculate the amount of periodic signals, it is difficult to determine threshold problems, especially for mobile real-time ECG monitoring, requiring low computational complexity and high detection accuracy, so the optimized wavelet processing is an ideal choice [9, 10].

However, two problems remain unresolved: firstly, which layer is more appropriate for feature detection after wavelet transform and secondly, whether the high-pass coefficient or low-pass coefficient is appropriate for feature location. If these key parameters are decided only by experiences, it is difficult to obtain systematic and scientific conclusions by experiments and emulations [11, 12]. This study investigated a detection method, which involved directly catching the signal frequency component during wavelet transform according to the frequency characteristics for different wavebands of ECG signal, to accurately locate the eigenvalue during wavelet transform. Currently, detection algorithms are mainly aimed at location and extraction of the QRS eigenvalue. Using these results and further reversing the electrophysiological activity of myocardial cells will be of great significance to automatic analysis and diagnosis of the physiological status of the heart [13]. Based on the eigenvalue detection, this research further studied the reverse analysis of myocardial action potential to enable automatic detection and diagnosis of heart diseases such as myocardial ischemia and heart failure.

## 2. Specific Frequency Coefficient Obtained by Wavelet Bandpass Filtering

A wavelet transform was performed for signal *f*(*x*) with frequency *P*_0_, where the high-pass component frequency was [*P*_0_/4, 3*P*_0_/4] and the low-pass component frequency was [0, *P*_0_/4] ∪ (3*P*_0_/4, *P*_0_). The high-pass filter *G*(*ω*) ≈ 2*A*_0_*e*^−*jNw*/2+*Nπ*/2^(sin⁡ (*w*/2))^*N*^ and low-pass filter *H*(*ω*)≈−2*A*_0_*e*^*jNw*/2^(cos⁡ (*w*/2))^*N*^ have two intersections in [0,2*π*]: (*π*/4, *G*(*π*/4)), (5*π*/4, *G*(5*π*/4)). The two intersections represent the region where low frequency transitions to high frequency. According to the Fourier convolution theorem, it can be concluded that the role of *G*(*ω*) and *H*(*ω*) on signal is equivalent to the transfer function in filtering circuit analysis. For further analysis of the suppression multiple of signals at the two critical points, $G(\pi /4)\approx \left\|2A_{0}e^{-N\left(j\pi /8-\pi /2\right)}(\sin\;\left(\pi /8\right){)}^{N}\right\|=2A_{0}{\left(\sin\;\left(\pi /8\right)\right)}^{N}=2A_{0}{\left(\sqrt{2-\sqrt{2}}/2\right)}^{N}$; for *A*_0_ = *H*_  _*N*__^(*N*)^(1)/*N*!, |*H*(*z*)|^2^ + |*H*(−*z*)|^2^ = 1, so *H*(*z*) ≤ |*H*(*z*)|^2^ ≤ 1, obviously *A*_0_ ≤ 1/*N*!, and then $G\left(\pi /4\right)\approx 2A_{0}{\left(\sqrt{2-\sqrt{2}}/2\right)}^{N}\leq \left(2/N!\right){\left(\sqrt{2-\sqrt{2}}/2\right)}^{N}$. Clearly, it is a function related to *N* with faster convergence rate. If *N* = 4, *G*(*π*/4) ≈ 0.00198178, and if *N* = 6, *G*(*π*/4) ≈ 0.0000101872. For a given positive *ε* close to 0, there exists *N*_0_ that always makes $\lim_{N\to N_{0}}\left(\left(2/N!\right){\left(\sqrt{2-\sqrt{2}}/2\right)}^{N}-\varepsilon \right)=0$. Then an appropriate vanishing moment can make the suppression multiple at the critical point infinitely small and thus make the extra-regional gains of signal passing this point close to 0, theoretically equivalent to cut-off state. Assume the signal sampling frequency is *P*_0_, including the noise with frequency of *P*_*s*_ = *P*_0_/2. Assume the wave-trapped and denoising tolerable frequency bandwidth is [*P*_*s*_ − Δ*P*, *P*_*s*_ + Δ*P*], where Δ*P* is frequency bandwidth increment. If the signal section is [*P*_1_, *P*_2_], after each wavelet transform, the high-pass component covers the frequencies of ((3*P*_1_ + *P*_2_)/4, (*P*_1_ + 3*P*_2_)/4), while the low-pass component covers the frequencies of [*P*_1_, (3*P*_1_ + *P*_2_)/4] ∪ [(*P*_1_ + 3*P*_2_)/4, *P*_2_]. For higher orders of filter for wave trapping, the overlaying area of high-pass frequency and low-pass frequency is smaller, the filter frequency curve is steeper, and the energy is more concentrated. To facilitate calculation, this study adopted normalised frequency as the unit: for the normalised frequency *R* in [*P*_1_, *P*_2_], the actual frequency *P*_*t*_ refers to

(1) $$\begin{array}{c} R=\frac{P_{t}-P_{1}}{P_{2}-P_{1}}. \end{array}$$

Any frequency range [*P*_1_, *P*_2_], after normalised processing, can be expressed in [0,1] solid area number field. According to the Shannon Theory, the sampling frequency should not be less than two times the maximum frequency in the analog signal frequency spectrum, so when directly filtering the sampling signal, the normalised frequency *R* should be in [0,0.5], while for filtering at the layer of second or above, *R* should be in [0,1].

Assume the normalised frequency for signal *S* is in [0, *P*_*t*_], and for the normalised frequency *P*_*s*_ ∈ [0, *P*_*t*_], if *P*_*s*_*∗*2^*N*^ ≈ 0.5 ± Δ*P*, the signal of frequency section *D* can be extracted from [*P*_1_, *P*_2_] by bandpass filtering after *N* wavelet transform. As a demonstration, a wavelet transform is performed for signal *S*, where *S*_*D*_ refers to the low-pass component after transform and *S*_*H*_ refers to the high-pass component after transform. According to the discussed situations, the following operations can be made according to concrete situations:

 
0.5 − Δ*P* ≤ *P*_*s*_ ≤ 0.5 + Δ*P* indicates *D*⊆*S*_*D*_, where the result *D*_*H*_ is returned, and then the algorithm ends.
 
*P*_*s*_ < 0.25 indicates *D*⊆*S*_*D*_ and a wavelet transform is performed for signal *S*_*D*_. *P*_*s*_ = 2*∗P*_*s*_, Δ*P* = 2*∗*Δ*P*; then this algorithm is repeated.
 
0.25 < *P*_*s*_ < 0.75 indicates *D*⊆*S*_*H*_, and a wavelet transform is performed for signal *S*_*D*_. *P*_*s*_ = 2*∗*(*P*_*s*_ − 0.25), Δ*P* = 2*∗*Δ*P*; then this algorithm is repeated.

## 3. Eigenvalue Extraction of QRS Wave Group and T Wave

How to accurately locate QRS wave group and T wave and extract their eigenvalues is of great significance for the detection of ECG eigenvalues. Affected by EMG interference, power frequency interference, and electromagnetic interference and noises, ECG signals are mixed with baseline drift and various noises, causing difficulties in the accurate location of ECG eigenvalues [14]. The basic method is to first analyze the frequency features of QRS wave group and extract the frequency components during wavelet decomposition, then enhance the signals according to certain strategy, and finally accurately locate the QRS wave group and T wave.

### 3.1. Analysis of Frequency Features of QRS Wave and T Wave

**Figure 1:**
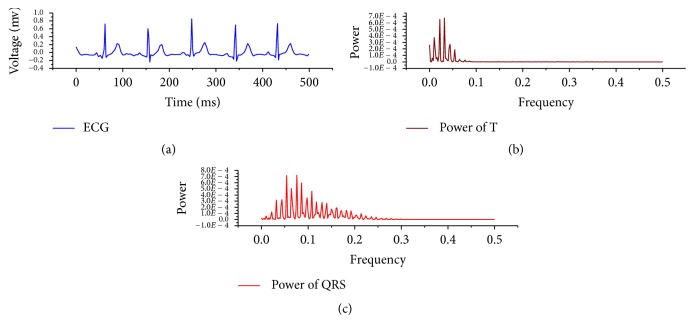
Schematic diagram of frequency characteristics of QRS wave and T wave of ECG signal. (a) shows the original ECG, (b) shows energy distribution of T wave after FFT, and (c) shows energy distribution of QRS wave after FFT.

Figure 1 shows the energy distribution of QRS wave and T wave on the frequency spectrum. It shows that the bandwidth for QRS wave is 0–40 Hz, accumulating nearly 99% of energy. To extract the wavelet system of QRS wave by bandpass filtering, the frequency bandwidth should be limited to about 20 Hz, so that the frequency section of 20 Hz bandwidth with maximum energy density in 0–40 Hz is achieved. By assuming *φ*(*t*) only covers QRS wave signals, *F*(*ω*) = ∫_−*∞*_^*∞*^*φ*(*t*)*e*^−*iωt*^*dt* can transform *φ*(*t*) from time domain to frequency domain. Section *D* of 10 Hz bandwidth with maximum energy density is calculated by the following formulae:

(2) Ps∫ss+10F2ωdω=∫ss+10∫−∞∞φte−iωtdt2dω,Ds,s+20 ∣ max⁡Ps∧s∈0,20.

Through calculation, it can be concluded that 76% of total energy is accumulated near 9.4 Hz–19.4 Hz of QRS wave. It can utilise the bandpass to extract the signal of this frequency section, where after the signal is enhanced, amplified, and processed, the higher identification can guarantee the signal is accurately locked. The wavelet coefficient has both frequency features and time domain features; location and extraction can be further made on the time domain. As shown in Figure 1, T wave has nearly 94% of energy in the 0–8 Hz frequency section. The bandwidth is narrower, to avoid overlapping with the baseband, and it selects 5 Hz waveband for extraction to calculate the frequency section of T wave of 5 Hz bandwidth with maximum energy density. By assuming *φ*(*t*) only covers T wave signal, *F*′(*ω*) = ∫_−*∞*_^*∞*^*ϕ*(*t*)*e*^−*iωt*^*dt*. Section *D*′ of 5 Hz bandwidth with maximum energy density is calculated by the following formulae:

(3) P′s∫ss+5F′2ωdω=∫ss+5∫−∞∞ϕte−iωtdt2dω,D′s,s+5 ∣ max⁡P′s∧s∈0,5.

Through calculation, it can be concluded that 75% total energy is accumulated near 3.0–8.0 Hz.

### 3.2. Extraction of Wavelet Coefficient Related to Features of QRS Wave Group and T Wave

According to the previous discussion, the wavelet signal component of QRS wave group should be extracted from 9.4 Hz to 19.4 Hz. To allow calculations, 10–20 Hz is used as the signal sampling section. When the sampling frequency is 200 Hz, the corresponding normalised frequency section is *D* = [0.05,0.10]. By analysis, the wavelet bandpass filtering algorithm flow of *D* = [0.05,0.10] is as follows.

The normalised frequency for the QRS frequency spectrum center is *p*_*m*_ = 0.075. When *p*_*m*_ < 0.25, the bandpass extraction fails to be made at the current wavelet decomposition layer, so the next round of wavelet transform needs to be made for the low-pass component after wavelet transform to finish the bandpass extraction. At this time, the resolution of wavelet-based signal space is shortened to a half, so the bandpass space should be expanded: *D* = 2*D* = [0.10,0.20], *p*_*M*_ = 2*∗p*_*M*_ = 0.15. In the second round of wavelet transform, *p*_*M*_ = 0.24 is included in the low-pass space *p*_*D*_ = (0,0.25) and the low-pass signal includes the direct current signal, so it should be further separated; let *p*_*M*_ = 0.48, to make the high-frequency component of the third round of wave transform as the extraction signal of QRS wave.

**Figure 2:**
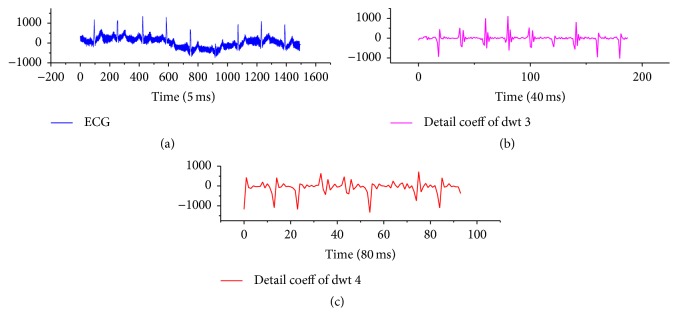
Schematic diagram of wavelet component of QRS wave and T wave of ECG signal. (a) shows the original ECG, (b) shows wavelet component of QRS wave after bandpass extraction, and (c) shows the wavelet component of T wave after bandpass extraction.

The energy distribution for T wave is 3.0–8.0 Hz, where the corresponding normalised frequency is [0.015,0.04] and its center frequency is *p*_*M*_ = 0.0275. After the fourth wavelet transform, *p*_*M*_ = 2^4^*∗p*_*M*_ = 0.44, and the corresponding frequency range is *D* = [0.24,0.64], *D* ⊂ *p*_*D*_, so it can select the high-frequency component after fourth filtering as the extraction signal, as shown in Figure 2.

### 3.3. Location and Eigenvalue Extraction of QRS Wave Group and T Wave

This processing has extracted the wavelet coefficient concentrating energies of the R wave signal, so next the coefficient can be accurately localised at the time domain. This study utilised a 0-1 extraction function *Z*(*t*) to transform the wavelet feature component *χ*(*t*) into a series of 0-1 square waves and then took the midpoint of each wave 1 as the time domain location result. The extraction function *Z*(*t*) is expressed as follows:

(4) $$\begin{array}{c} Z\left(\;t\;\right)=\left\{\begin{array}{cc} 0 & \overset{L}{\underset{k=0}{\sum }}\chi^{2}\left(t-k\right)<\frac{M}{2},\\ 1 & \overset{L}{\underset{k=0}{\sum }}\chi^{2}\left(t-k\right)\geq \frac{M}{2}. \end{array}\right. \end{array}$$

During the algorithm implementation process, the threshold value *M* should be upgraded:

(5) if: ∑k=0Lχ2t−k≥M2then: M=M1−λ+λ∑k=0Lχ2t−k.

**Figure 3:**
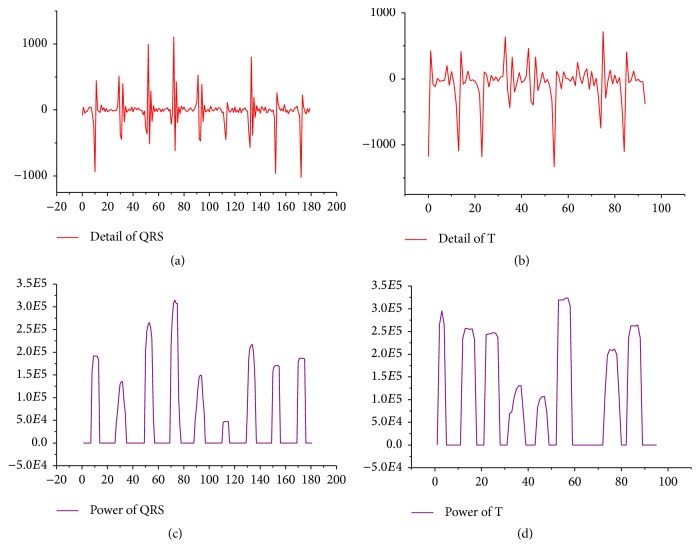
Schematic diagram of feature location of QRS wave and T wave of ECG signal. (a) shows QRS component, (b) shows T wave component, (c) shows QRS energy, and (d) shows T wave energy.

**Figure 4:**
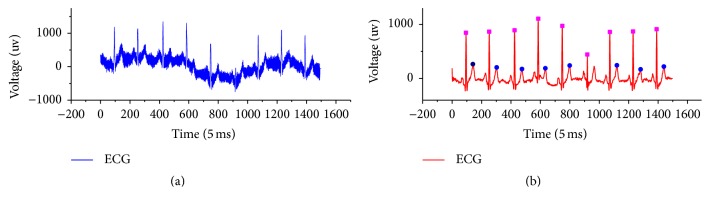
Schematic diagram of feature detection of QRS wave and T wave. (a) shows the original ECG signal and (b) shows the features location of ECG filtering.

Generally, a real number of *λ* ≤ 0.25 is used. While the *L* value can be determined as per the actual width of R wave in the time domain, generally speaking, if R wave lasts for *T*, *L* = *T*/2. When the square wave is wider, it should be further localised to minimum time. A trigger mechanism should be set so that when *Z*(*t*) = 1, *Q* = *t* is triggered, and when *Z*(*t*) changes from 1 to 0, the calculation and location will be made by *Q* = (*Q* + *t*)/2. A concrete algorithm implementation can be finished in a loop iteration where the time complexity is *O*(*n*).  Figure 3 shows the location of features of QRS wave and T wave, and Figure 4 shows the location results of algorithm features. The feature detection and location are based on the wavelet transform and are combined with signal denoising, compressing, and other processing so that the algorithm can save resources.

## 4. ECG Reverse Analysis and Myocardial Membrane Action Potential Feature Detection

The electrocardiogram (ECG) is a dynamic potential difference of myocardial membrane action potential between two points of body surface, and it can objectively reflect the physiological status of the heart [15]. ECG is of great significance to clinical diagnosis, but ECG automatic disease diagnosis and analysis face certain technology challenges. Some studies [16, 17] have put forward an ECG mode recognition method to establish a complete ECG feature template database in advance and then match it with extracted signal for analysis. However, this method has difficulties in establishing a complete ECG template and complexity in matching analysis time; it is not suitable for mobile and real-time ECG. Other studies [18, 19] have put forward an artificial intelligent algorithm, by learning, training, and accumulating the knowledge and experiences to perform intelligent recognition on extracted signals. This method can adapt to big-data and high-performance platform processing but is of insufficient resources for mobile and real-time ECG. The current study reversely calculated the heart outer membrane potential and obtained its eigenvalue based on previously extracted ECG eigenvalue, to enable the physiological status of heart to be shown and to provide a basis for automatic analysis of heart disease diagnosis and health surveillance.

### 4.1. Heart Membrane Potential Action Figure and Feature Model

**Figure 5:**
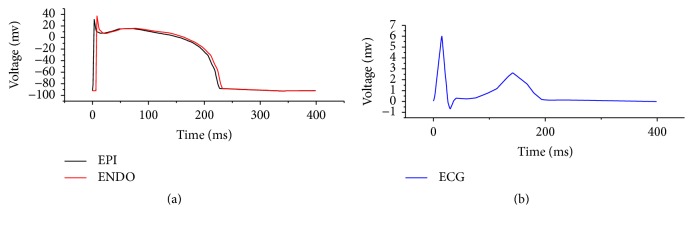
Relationship between myocardial membrane potential and ECG signal. (a) shows the myocardial membrane potential and (b) shows the original ECG signal.

The potential difference between the inside and outside of the myocardial membrane is called the transmembrane potential or membrane potential. When the myocardial cells are excited by irritation, the membrane potential will suddenly change; the potential inside the membrane will change from negative potential to positive potential [20], while the potential outside the membrane will change from positive potential to negative potential. This change in myocardial transmembrane potential is called action potential.  Figure 5 shows the relationship between myocardial membrane potential and ECG signal. The TNNP model is the single cell transmembrane potential action model presented by Köhler et al. [21]. By H-H equivalent model principle, the cell membrane acts as a capacitor, the ionic currents and pumps are equivalent to interrelated power and resistance [22], so the single cell electrophysiological model of TNNP model can be expressed by

(6) $$\begin{array}{c} \frac{dV_{m}}{dt}=-\frac{I_{ion}+I_{stim}}{C_{m}}, \end{array}$$

where *V*_*m*_ is membrane potential, *t* is time, *I*_stim_ is outside stimulated current, and *C*_*m*_ is unit membrane capacitance. *I*_ion_ as total transmembrane current can be expressed by

(7) Iion=INa+IKI+Ito+IKr+IKs+ICaL+INaCa+INaK+IpCa+IpK+IbCa+IbNa,

where *I*_CaL_ is type-L Ca^2+^ current, *I*_NaCa_ is the current of Na^+^/Ca^2+^ exchanger, *I*_NaK_ is Na/K pump current, *I*_pCa_ and *I*_pK_ are calcium and potassium current at platform phase, respectively, *I*_bCa_ and *I*_bK_ are background potassium and calcium current, respectively, *I*_Na_ is rapid Na^+^ current, *I*_KI_ is inward rectifier K^+^ current, *I*_to_ is transient outward current, *I*_Kr_ is rapid delayed rectifier K^+^ current, and *I*_Ks_ is slow delayed rectifier K^+^ current. The H-H model makes the influence factor of each current equivalent to a control logic gate to show the electrophysiological status of heart cells. The control parameters of the membrane potential action equation are as many as 256 [23]. For ECG automatic detection and calculation, the detection and extraction of large amounts of fine and sensitive physiological parameters is a complicated and difficult task, not suitable for a mobile and real-time calculation platform.

This complicated heart potential action equation cannot be directly determined by parameters and is hard to fit with polynomials. Based on this, the current study utilised big data to establish a standardised heart outer membrane action potential mode and modulated this model with simple parameters, to enable this model to show different heart outer membrane action potential characteristics and reversely calculate this model with ECG eigenvalue. The myocardial cell action potential can directly reflect the electrophysiological activity of cells in universality and stability; under normal circumstances, it can better show the electrophysiological activity status of myocardial cells. This study used the heart outer membrane potential database as sample data to perform regression analysis and establish the v-lead left and right standard models, where the corresponding figures are as follows.

Let *C*_*L*_(*t*) indicate left epimyocardium standard action potential of human and let *C*_*R*_(*t*) indicate right epimyocardium standard action potential of human.

*F*(*R*, *t*, *k*, *h*_*R*_) = *h*_*R*_*C*_*R*_(*kt*) means the *x*-coordinate of *C*_*R*_(*t*) is scalable in *k* times and the *y*-coordinate is scalable in *h*_*R*_ times.

**Figure 6:**
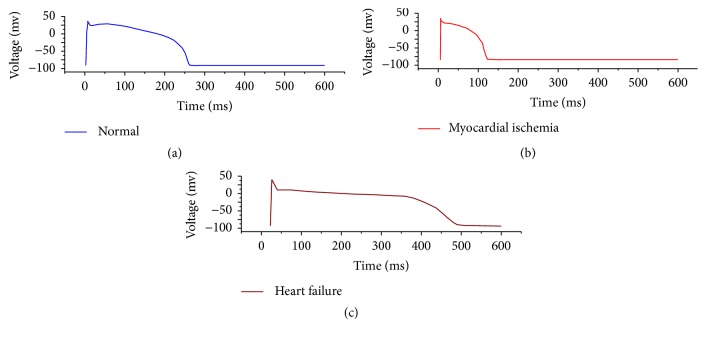
Comparison of normal and abnormal epicardium action potentials. (a) shows the normal epicardium action potential, (b) shows the action potential of epicardium with myocardial ischemia, and (c) shows the action potential of epicardium with heart failure.

*F*(*L*, *t*, *k*, *h*_*L*_) = *h*_*L*_*C*_*L*_(*kt*) means the *x*-coordinate of *C*_*L*_(*t*) is scalable in *k* times and the *y*-coordinate is scalable in *h*_*L*_ times.  Figure 6 compares the normal and abnormal epicardium action potentials, showing *k*, *h*_*L*_, and *H*_*R*_ have influences on epicardium action potential forms.  Figure 6(b) shows the myocardial action potential figure and ECG with myocardial ischemia, and Figure 6(c) shows the myocardial action potential figure and ECG with heart failure. In the case of myocardial ischemia, in *F*(*R*, *t*, *k*, *h*_*R*_), *k* is less than 0.9 and *H*_*R*_ is less than 0.95. In the case of heart failure, in *F*(*R*, *t*, *k*, *h*_*L*_), *k* is less than 1.1 and *h*_*L*_ is less than 0.96. Therefore, eigenvalues *k* and *h*_*L*_ can effectively show the status of myocardial electrophysiology activity and thus provide a way for intelligent diagnosis and analysis.

### 4.2. Extraction of Potential Features of Heart Membrane

Through detection of ECG eigenvalue, it can obtain basic features of the ECG signal: to locate the time of R peak value and T peak value, obtain R peak value Ψ and T peak value *T*, and acquire the integral of ECG signal time on time Δ from R wave starting time to rest time. Accordingly, the following simultaneous equations model can be established:

(8) Y1=FR,tT−tR,k,hR,Y2=FL,tT−tR,k,hL,T=hRY1−hLY2,Δ=khRΦ1−hLΦ2,

where Φ_1_ and Φ_2_ indicate the integral of *F*(*R*, *t*, *k*, *h*_*R*_) on time *t*. This model is unidentifiable from structure. To simplify calculations, this study assigned 1, respectively, to *r*  *k*, *h*_*R*_, *h*_*L*_ to get *Y*_1_, *Y*_2_, and hence, formula (7) is simplified into a regression simultaneous equations model:

(9) T=hRY1−hLY2,Δ=khRΦ1−hLΦ2.

In the simultaneous equations model, the first equation is a linear equation with two unknowns and is not dependent on the second equation, so the least square method can be adopted independently for unbiased estimation of parameters *h*_*R*_, *h*_*L*_. Through detection and analysis, the experimental data can be attained:

(10) T=T1,T2,…,Tn,Y1=Y11,Y12,…,Y1n,Y2=Y21,Y22,…,Y2n.

The corresponding deviation equation is

(11) $$\begin{array}{c} \sum {v_{i}}^{2}=\sum {\left[\;T_{i}-\left(\;h_{R}Y_{1i}+h_{L}Y_{2i}\;\right)\;\right]}^{2}. \end{array}$$

Calculate the partial derivative of *h*_*R*_, *h*_*L*_, let it be 0, and then solve the equation to get

(12) hR=s1y·s22−s2y·s12s11s22−s122,hL=s11·s2y−s12·s1ys11s22−s122,

where

(13) s11=∑Y1i2−∑Y1i2n,s22=∑Y2i2−∑Y2i2n,s12=∑Y1iY2i+∑Y1i∑Y2in,s1y=∑Y1iTi−∑Y1i∑Tin,s2y=∑Y2iTi−∑Y2i∑Tin.

By *m* rounds of the least square method, the estimated values of *h*_*R*_, *h*_*L*_ of *m* sets will be attained:

(14) hR=hR1,hR2,…,hRm,hL=hL1,hL2,…,hLm.

**Figure 7:**
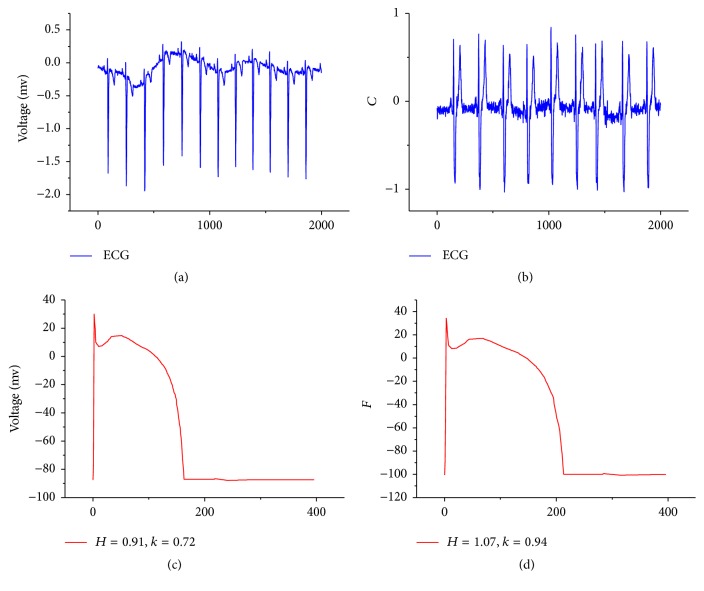
Schematic diagram of ECG reverse features analysis results. (a) shows the ECG with myocardial ischemia, (b) shows the normal ECG, (c) shows the reverse calculation results of (a), and (d) shows the reverse calculation results of (b).

Substitute them into Δ = *k*(*h*_*R*_Φ_1_ − *h*_*L*_Φ_2_) for regression fitting of the least square method on *k*. Generally speaking, the system will tend to be stable after multiple rounds of iterations, and then the parameters *h*_*R*_, *h*_*L*_, *k* can objectively reflect the basic features of myocardial electrical activity.  Figure 7 shows ECG reverse feature analysis results.

**Figure 8:**
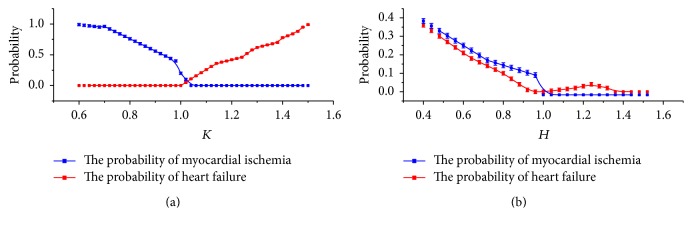
Schematic diagram of the correlation between *K* and *H* and myocardial ischemia and heart failure. (a) shows the *K* correlation and (b) shows the *H* correlation.

This investigation sampled 15 records, respectively, from the European ST-T database and the BIDMC congestive heart failure database and sampled 10 records from ECG ID for comparative experiments, to analyze the correlation between *K* and *H* and myocardial ischemia and heart failure. When *K* is less than 0.85, the myocardial ischemia probability begins to increase; when *K* is less than 0.7, the probability is as high as 89%. When *K* is greater than 1.2, the correlation of heart failure will obviously increase; when *K* is greater than 1.45, the probability of heart failure will be as high as 77.5%. The correlations are shown in Figure 8.

## 5. Experiment and Emulation Results

This research utilised the ECG ID database to evaluate the ECG detection method. The ECG database includes the two-channel ECG records of 48.5 hours, with 11 digits of resolutions and 10 mV. ECG records from this database cover sharp waves and high T waves, negative QRS wave group, small QRS wave group, wide QRS wave group, myoelectricity noise, baseline drift, sudden change of QRS amplitude, QRS morphological mutation, polymorphous premature ventricular contraction, long pause, and irregular heart rhythm. Detection and experiments were performed by the algorithm presented in this paper to obtain six quantitative results: correct detection of R or T peak time showed true positive (TP); loss of R or T peak time showed false negative (FN); and the noise spike detected to be R or T peak time showed false positive (FP). To evaluate the performance of the given detection algorithm, the following formulae should be utilised to calculate the sensitivity (Se) and detection error rate (DER). To evaluate the detection accuracy and accuracy rate of this method, accuracy (Acc) is defined. In Table 1, R peak detection rate of first channel (each) of 16 ECG records in the MIT-BIH arrhythmia database is summarised. By running the algorithm for detection, QRS wave totally generates 43 FN pulses and 44 FP beats, totalling 87 failures. The detection accuracy of ECG records change from 99.32% to 100% based on normal and pathological ECG signal features and different noises. The detection accuracy for QRS wave is slightly higher than T wave; T wave totally generates 48 FN pulses and 54 FP beats, totalling 102 failures, as shown in Table 1:

(15) Se=TPTP+FN×100%,DER=FP+FNTP×100%,Acc=TPTP+FP+FN×100%.

To test the detection effects of the algorithm on pathological ECG signals of myocardial ischemia and heart failure, this study used 15 ECG samples with myocardial ischemia from the European ST-T database, 15 ECG samples with heart failure from the BIDMC congestive heart failure database, and 10 raw signal ECG samples from the ECG ID database, with 100 heart rhythm signals included for each sample. As seen from the detection results in Table 2, 14 ECG samples with myocardial ischemia were accurately detected, with an accuracy of 93.3%; 13 samples with heart failure were accurately detected, with an accuracy of 86.7%; and 10 raw signal ECG samples were accurately detected, with an accuracy of 100%.

Signal analysis and processing can be divided into two methods: direct analysis and transformation analysis. Signal transformation analysis and processing is carried out by mapping the signal to another domain, such as wavelet transformation or EMD transformation.

**Figure 9:**
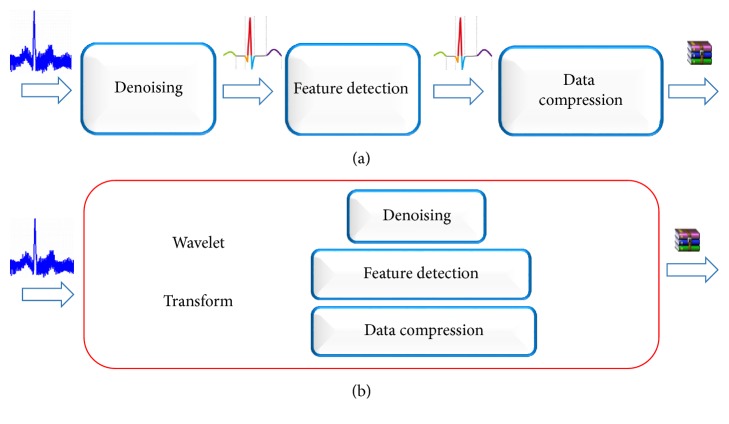
Comparison of two different algorithms for ECG signal processing.

Figure 9(a) shows the ECG signal processing of conventional processes, including denoising, feature detection, and filtering of the three main processes. If *n* is the length of the signal under normal circumstances, each process needs to undergo a transformation, assuming that each transformation requires a time frequency of *T*_0_(*n*) = *C*_0_*n*^2^. Under these conditions, denoising, detection, and compression are performed. Assuming that the time frequency of each process is *T*_1_(*n*) = *C*_1_*n*, the total time required for the conventional signal processing method is 3(*T*_0_(*n*) + *T*_1_(*n*)) = 3(*C*_0_*n*^2^ + *C*_1_*n*). If the proposed algorithm is used for completing the denoising, the detection, and the compression operation, the whole process only needs to perform the wavelet transform one time, with a total time taken of *T*_0_(*n*) + 3*T*_1_(*n*) = *C*_0_*n*^2^ + 3*C*_1_*n* (as shown in Figure 9(b)). The algorithm proposed in this paper can reduce the time for transformation between signal domains due to the combination of feature detection, signal filtering, and signal compression, thus saving resources and speeding up the operation. As the wavelet transformation needs to undergo both processes of decomposition and reconstruction, the useful signal is often lost in the process of conversion; for example, the symmetry of the orthogonal wavelet will decrease with the increase of the order of the vanishing moment and the process will cause signal distortion. In this paper, we present a method which reduces the time for transformation, so useful signal loss can be reduced and accuracy of detection increased.

## 6. Conclusion

The wavelet transform was performed to achieve wave trapping extraction, to extract the feature signal component from wavelet decomposition signal and then enhance and locate the energy expressing eigenvalue. This method can integrate the feature location, signal filtering, signal compressing, and other processing, and therefore it can save computing resources, speed up the processing, and enhance the detection accuracy. This study utilised ECG eigenvalue to reversely calculate myocardial potential action features. This research also established the simultaneous equations model to represent the myocardial membrane potential activity and utilised iterative regression to analyze the asymptotic approximation, to cause the model to accurately show the myocardial potential action and provide the basis for automatic diagnosis of heart diseases.

## Figures and Tables

**Table 1:** Performance evaluation on R and T waves by using first channel of MIT-BIH arrhythmia database.

ECG record	Total (beats)	FN_R (beats)	FN_T (beats)	FP_R (beats)	FP_T (beats)	DER_R (%)	DER_T (%)	Se_R (%)	Se_T (%)	Accuracy_R (%)	Accuracy_T (%)
200	2601	1	1	6	8	0.27	0.35	99.96	99.96	99.73	99.65
201	1963	0	1	2	2	0.10	0.15	100.00	99.95	99.90	99.85
202	2136	1	2	7	9	0.38	0.52	99.95	99.91	99.63	99.49
203	2980	11	12	5	6	0.54	0.61	99.63	99.60	99.46	99.40
205	2656	1	1	0	1	0.04	0.08	99.96	99.96	99.96	99.92
207	1862	0	1	1	1	0.05	0.11	100.00	99.95	99.95	99.89
208	2955	8	9	5	5	0.44	0.48	99.73	99.69	99.56	99.53
209	3005	1	1	0	1	0.03	0.07	99.97	99.97	99.97	99.93
210	2650	6	7	3	4	0.34	0.42	99.77	99.74	99.66	99.58
215	3363	0	1	0	0	0.00	0.03	100.00	99.97	100.00	99.97
217	2208	2	2	2	3	0.18	0.23	99.91	99.91	99.82	99.77
219	2154	1	0	2	2	0.14	0.09	99.95	100.00	99.86	99.91
220	2048	1	1	1	1	0.10	0.10	99.95	99.95	99.90	99.90
223	2605	2	1	0	0	0.08	0.04	99.92	99.96	99.92	99.96
228	2053	7	7	7	8	0.69	0.74	99.66	99.66	99.32	99.27
230	2256	1	1	3	3	0.18	0.18	99.96	99.96	99.82	99.82

**Table 2:** Detection results of 40 ECG pathological samples.

ECG record	*K*	*H*_*R*	*H*_*L*	Clinical signs	Detection result
E0111	0.71	0.97	0.95	Myocardial ischemia	TP
E0112	0.79	0.89	0.87	Myocardial ischemia	TP
E0113	0.69	0.91	0.93	Myocardial ischemia	TP
E0114	0.63	0.93	0.92	Myocardial ischemia	TP
E0115	0.96	0.99	0.91	Myocardial ischemia	FP
E0116	0.71	0.88	0.92	Myocardial ischemia	TP
E0117	0.73	0.89	0.88	Myocardial ischemia	TP
E0118	0.71	0.91	0.87	Myocardial ischemia	TP
E0112	0.69	0.91	0.93	Myocardial ischemia	TP
E0113	0.63	0.93	0.92	Myocardial ischemia	TP
E0114	0.66	0.89	0.91	Myocardial ischemia	TP
E0115	0.71	0.88	0.92	Myocardial ischemia	TP
E0116	0.70	0.86	0.88	Myocardial ischemia	TP
E0117	0.79	0.87	0.87	Myocardial ischemia	TP
E0118	0.63	0.91	0.93	Myocardial ischemia	TP
Chf01	1.79	0.81	0.83	Heart failure	TP
Chf02	1.62	0.79	0.81	Heart failure	TP
Chf03	1.16	0.91	0.97	Heart failure	FN
Chf04	1.70	0.86	0.88	Heart failure	TP
Chf05	1.69	0.79	0.86	Heart failure	TP
Chf06	1.79	0.87	0.83	Heart failure	TP
Chf07	1.62	0.79	0.81	Heart failure	TP
Chf08	1.66	0.81	0.77	Heart failure	TP
Chf09	1.10	0.92	0.98	Heart failure	FN
Chf10	1.69	0.99	0.86	Heart failure	TP
Chf11	1.79	0.89	0.83	Heart failure	TP
Chf12	1.62	0.89	0.81	Heart failure	TP
Chf13	1.65	0.81	0.77	Heart failure	TP
Chf14	1.71	0.82	0.88	Heart failure	TP
Chf15	1.68	0.79	0.86	Heart failure	TP
Person_01/rec_1	1.01	0.98	0.99	Normal	TP
Person_01/rec_1	1.02	1.04	1.01	Normal	TP
Person_01/rec_1	0.99	1.02	0.97	Normal	TP
Person_01/rec_1	1.01	0.97	0.99	Normal	TP
Person_01/rec_1	1.05	0.99	1.02	Normal	TP
Person_01/rec_1	1.01	0.98	0.99	Normal	TP
Person_01/rec_1	1.04	0.98	0.99	Normal	TP
Person_01/rec_1	1.02	1.01	1.01	Normal	TP
Person_01/rec_1	0.99	1.02	0.97	Normal	TP
Person_01/rec_1	1.01	0.97	0.99	Normal	TP
Person_01/rec_1	1.00	0.98	1.02	Normal	TP
